# Detection of Telomeric DNA:RNA Hybrids Using TeloDRIP-qPCR

**DOI:** 10.3390/ijms21249774

**Published:** 2020-12-21

**Authors:** Ilaria Rosso, Fabrizio d’Adda di Fagagna

**Affiliations:** 1 IFOM—The FIRC Institute of Molecular Oncology, 20139 Milan, Italy; 2 CNR—Consiglio Nazionale delle Ricerche, Istituto di Genetica Molecolare, 27100 Pavia, Italy

**Keywords:** telomeres, DNA:RNA hybrids, DNA:RNA immunoprecipitation

## Abstract

Because of their intrinsic characteristics, telomeres are genomic loci that pose significant problems during the replication of the genome. In particular, it has been observed that telomeres that are maintained in cancer cells by the alternative mechanism of the lengthening of telomeres (ALT) harbor higher levels of replicative stress compared with telomerase-positive cancer cells. R-loops are three-stranded structures formed by a DNA:RNA hybrid and a displaced ssDNA. Emerging evidence suggests that controlling the levels of R-loops at ALT telomeres is critical for telomere maintenance. In fact, on the one hand, they favor telomere recombination, but on the other, they are a source of detrimental replicative stress. DRIP (DNA:RNA immunoprecipitation) is the main technique used for the detection of R-loops, and it is based on the use of the S9.6 antibody, which recognizes preferentially DNA:RNA hybrids in a sequence-independent manner. The detection of DNA:RNA hybrids in repetitive sequences such as telomeres requires some additional precautions as a result of their repetitive nature. Here, we share an optimized protocol for the detection of telomeric DNA:RNA hybrids, and we demonstrate its application in an ALT and in a telomerase-positive cell line. We demonstrate that ALT telomeres bear higher levels of DNA:RNA hybrids, and we propose this method as a reliable way to detect them in telomeres.

## 1. Introduction

DNA replication is a fundamental process through which cells duplicate their genetic material in order to produce two daughter cells. The faithfulness of this mechanism is crucial in order to guarantee that the genetic information is preserved. In fact, defects in the replication of DNA are associated with cell death and human diseases. However, many obstacles challenge this process, causing the slowing down or the stalling of the replication fork. These events are commonly defined as replication stress, and require specific pathways to address them, before their persistence unleashes major deleterious effects [1].

Some genomic loci are particularly prone to experience replication stress because of their intrinsic characteristics. Among these, there are telomeres, long arrays of TTAGGG tandem repeats coated by shelterin proteins, which protect chromosome termini from signaling and fusions by folding into a lariat structure called a T-loop [2]. High order structures such as T-loops constitute a blockade to DNA replication machinery, and need to be dismantled to allow for efficient DNA duplication [3]. In addition, the G-rich sequence of telomeric DNA favors the formation of G-quadruplexe, secondary structures that, in turn, are a challenge for the replication process [4]. Moreover, the long non-coding RNA TERRA (telomeric repeat-containing RNA), which is transcribed from subtelomeres into the telomeric repeats, and is able to form hybrids pairing with the C-rich strand of telomeric DNA, causing further replication stress [5,6,7]. Together, these features make telomeres difficult-to-replicate loci.

The alternative lengthening of telomeres (ALT) pathway is a mechanism of telomere extension that 10–15% of human cancers use to guarantee telomere maintenance in the absence of telomerase reactivation [8]. It engages a number of pathways of non-canonical DNA synthesis, whose mechanistic details are yet to be fully elucidated. Emerging evidence connects ALT to replicative stress and DNA damage [9,10]. In fact, it has been shown that telomeric DNA:RNA hybrids play a double role—they are a source of replicative stress at telomeres, but they also promote homology-directed telomere elongation [7,11]. Therefore, their modulation needs to be finely regulated, and several ALT factors have been reported to attend to this critical task [7,12,13].

The increasing interest in the study of R-loops requires a reliable method to detect and map these structures in the genome. The main technique used for this kind of investigation is DRIP (DNA:RNA immunoprecipitation), which is based on the preferential affinity of the monoclonal antibody S9.6 for DNA:RNA hybrids, regardless of their sequence [14]. RNase H digestion of the samples prior to immunoprecipitation is an essential control that ensures that the detected signal represents a genuine hybrid. In fact, it is worth noting that this antibody displays a nanomolar affinity for dsRNA, and this needs to be taken into consideration when using S9.6 as a tool to detect R-loops, both by immunoprecipitation and by imaging techniques [15,16]. DRIP protocols come in many variants, each attempting to address some of the disadvantages of the basic method. Fragmentation of the chromatin prior to immunoprecipitation is usually carried out by restriction enzymes, but this approach is not optimal for those loci that do not carry restriction sites, such as telomeres, and their resolution is low [17]. Sonication can be used, but the increased resolution is counterbalanced by a decreased signal, as a result of the mechanical stress exerted on the chromatin. In fact, the energy provided by sonication may promote branch migration, leading to the loss of the RNA strand of the hybrid and to the re-annealing of the two DNA strands [18]. After immunoprecipitation, the material can be used either to prepare a library for sequencing, so as to have a broader view of the DNA:RNA hybrid landscape, or it can be analyzed by qPCR using target-specific primers [19]. More complex approaches can be adopted to get strand-specific information on which a filament of DNA is involved in the hybrid formation, such as Bis-DRIP, cDRIP, or ssDRIP [19,20,21]. Other protocols for the detection of R-loops include the use of catalytically inactive RNAse H, but the use of these methods is currently limited, because of their laboriousness [22,23].

Because of their repetitive nature, the study of DNA:RNA hybrids at telomeres poses additional challenges. Sonication is required, as the absence of restriction sites in telomeres makes it the only suitable approach to obtain short telomeric fragments. The detection of telomeric hybrids is usually carried out by amplifying the subtelomeric region of the TERRA molecules transcribed from single chromosomes using RT-qPCR [7,24]. This approach limits the analysis to a subset of telomeres, and is not focused on the hybrid formed at the telomeric repeats, but on the subtelomeric region. Alternatively, DRIP samples can be analyzed by dot-blot using a radioactive-labeled probe that recognizes telomeric sequences [12]. The hazards of radionuclides and the time needed for the blot, the hybridization, and the exposure, make this method risky and time consuming.

Here, we couple the DRIP protocol with the detection of telomeric repeats by qPCR, using a set of validated primers that allow for the accurate quantification of the telomeric content, despite its repetitive nature [25,26]. We validate TeloDRIP-qPCR by performing this protocol in two cell lines, we compare the obtained results with dot-blot detection, and we show that telomeric repeats in ALT cell line U2OS bear more hybrids compared with the telomerase-positive HeLa cell line. We therefore propose TeloDRIP-qPCR as a faster, safer, and more reliable method to detect DNA:RNA hybrids at telomeres.

## 2. Results

### 2.1. TeloDRIP-qPCR Protocol

To validate our protocol, we performed it on two different cell lines, namely: U2OS, a bone osteosarcoma cell line that elongates telomeres through ALT mechanisms, and HeLa, a telomerase-positive cervical carcinoma cell line. As the purpose of DRIP is to recover DNA:RNA hybrids that form in the genome, it is of paramount importance that the components of these structures are preserved. For this reason, careful manipulation of the sample in RNAse free conditions and the use of RNAses inhibitors are highly recommended.

#### 2.1.1. Chromatin Purification

The DRIP protocol is based on the immunoprecipitation of DNA:RNA hybrids from fragmented chromatin samples. The starting material is obtained from cell lysis and nucleic acid extraction by phenol/chloroform. The amount of cells used is based on the amount of chromatin needed to complete the protocol, and may vary from cell line to cell line. Specifically, around 10^7^ HeLa cells and 1.2 × 10^7^ U2OS cells are used. The thick chromatin suspension resulting from lysis can be difficult to pipette, therefore it is recommendable to split the cells in more than one tube (so to have around 2 × 10^6^ cells per lysis reaction) and pool the material after sonication. During precipitation in ethanol, the chromatin pellet is better spooled out and not centrifuged, so as to reduce RNA contaminations [19].

#### 2.1.2. Fragmentation of the Chromatin

**Figure 1:**
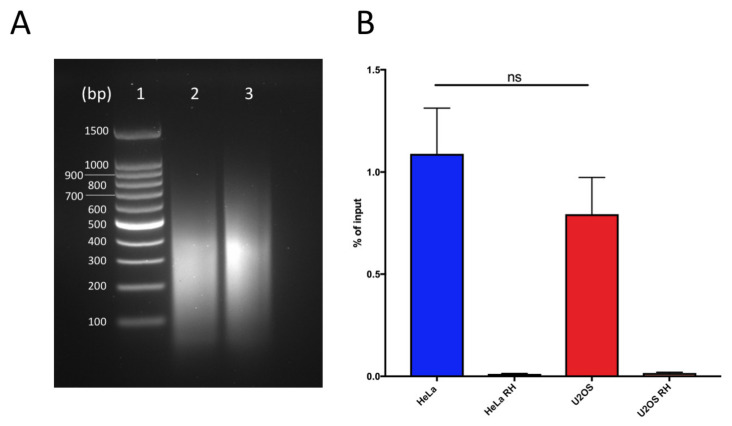
DNA:RNA immunoprecipitation successfully retrieves DNA:RNA hybrids: (**a**) Chromatin digestion profiles for HeLa (lane 2) and U2OS (lane 3). Lane 1 corresponds to DNA ladder 100 bp (Promega). (**b**) Quantification of DNA:RNA hybrids at RPL13 positive control locus in U2OS and HeLa cells as measured by qPCR. Bars and error bars are the averages and SEM of four experiments. *p*-values are calculated using one-way ANOVA (ns, not significant).

As the fragmentation method determines the size of the fragments, it is important for the level of resolution for the detection, and therefore it is a crucial point in the protocol setting. Despite the decreased yield caused by the mechanical stress, we prefer unbiased sonication to restriction digestion, as the latter does not allow for the fragmentation of telomeric repeats. We suggest finding conditions for each cell line that allow for obtaining short fragments (around 300 bp), while avoiding excessive sonicating and loss of signal of the hybrids (Figure 1A).

#### 2.1.3. RNAseH Control Digestion

Before immunoprecipitation, RNAse H treatment is performed as a negative control, so as to confirm the specificity of the hybrid signal. Then, 10 µg of fragmented chromatin is either treated with 20 U RNAse H or mock treated for 5 h at 37 °C at 350 rpm. In order to have enough material to proceed with the immunoprecipitation, four RNAseH reactions and four mock controls for each cell line are performed in parallel.

#### 2.1.4. Immunoprecipitation

For immunoprecipitation, 8 µg of chromatin is incubated overnight at 4 °C on a rotating wheel with 4 µg of S9.6 antibody (MABE 1095, Merck, Darmstadt, Germany). Four reactions can be performed in parallel for each condition, in order to generate sufficient amounts of material for dot-blot analyses. Before adding the antibody, 1/20 of the volume of the reaction is collected to be used as the input control.

#### 2.1.5. Elution

Samples are incubated with a mixture of Dynabeads protein A and protein G for 2 h at 4 °C on a rotating wheel, in order to collect the immunocomplexes that formed overnight. During elution, samples are first treated with 80 µg/mL RNAseA for 30 min at 50 °C at 350 rpm to remove any contribution from RNA, and then with 560 µg/mL proteinase K for 45 min at 55 °C at 750 rpm to remove immunocomplexes from the beads. After elution, DNA is precipitated. Phenol/chloroform extraction is avoided so as to prevent DNA loss [27]. Eluted samples can be analyzed by qPCR to confirm the success of the procedure by testing the genomic loci known for hybrid accumulation, such as RPL13A, as the positive controls (Figure 1B) [28].

### 2.2. ALT Cells Display Higher Levels of DNA:RNA Hybrids at Telomeric Repeats Compared to Telomerase Positive Cells

**Figure 2:**
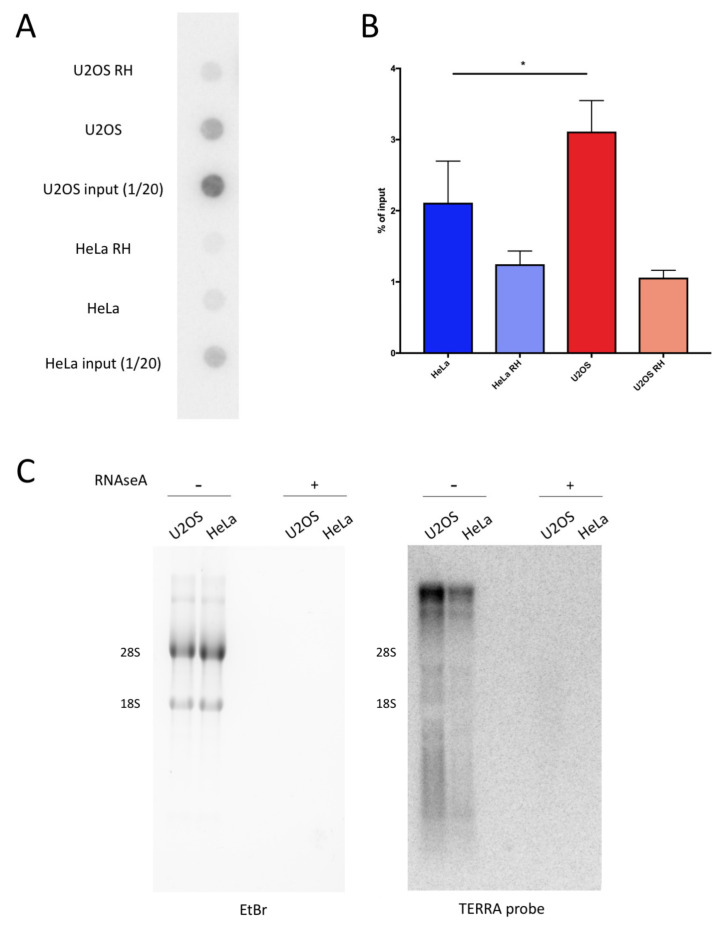
U2OS display higher levels of telomeric DNA:RNA hybrids compared with HeLa. (**a**) Dot-blot hybridization of DRIP (DNA:RNA immunoprecipitation) in HeLa and U2OS cells using radiolabeled G-rich telomeric oligonucleotides. (**b**) Quantification of experiment in (**a**). Bars and error bars are averages and SEM of four experiments. *p*-values are calculated using one-way ANOVA (* *p* < 0.05). (**c**) TERRA (telomeric repeat-containing RNA) Northern blot hybridizations of RNA from U2OS and HeLa pre-treated with RNaseA or left untreated. Ethidium bromide staining of agarose gel is shown as the control for loading.

We analyzed the DRIP samples from U2OS and HeLa ALT cells, which maintain the telomere length through the activity of the telomerase. Previous studies have shown that the modulation of R-loops at telomeres is critical for the ALT pathway [7,12]. Therefore, we tested if the telomeres in the U2OS ALT cells accumulate more hybrids than non-ALT HeLa cells. To this end, using blot-dot, we analyzed the DRIP experiments performed on U2OS and HeLa, and we hybridized the membranes with a radiolabeled DNA probe that recognizes the telomeric sequence (Figure 2A). Despite starting from the same amount of materials, for the input samples, the telomeric repeats were more abundant in the U2OS compared with HeLa cells. This was expected, as ALT cells have a longer mean telomere length compared with telomerase positive cells [29]. The DRIP signals from the dot-blot were calculated as the percentage of input signals, taking into account that the input corresponded to 1/20 of the DRIP reactions. U2OS cells displayed a higher percentage of telomeric repeats involved in hybrid formation compared with HeLa cells, indicating an increased presence of these structures at ALT telomeres (Figure 2B). In addition, we performed Northern blot analysis to assess the TERRA RNA levels in U2OS and HeLa. As shown previously, U2OS displayed increased levels of TERRA compared with HeLa [7]. This may be an important contribution to the prevalence of DNA:RNA hybrids at telomeres in U2OS with respect to HeLa.

### 2.3. TeloDRIP-qPCR Protocol is a Reliable Method for the Detection of Telomeric DNA:RNA Hybrids

The detection of telomeric DNA:RNA hybrids using dot-blot is a long procedure that requires overnight incubation with the telomeric probe and hours-long exposition of the membrane. Moreover, relatively high amounts of material are required in order to obtain a clear signal. Differently, the detection of hybrids at the RPL13 locus through PCR is fast and easy. Therefore, we decided to detect telomeric hybrids using qPCR. The detection of highly repetitive regions using qPCR is a challenge—the primers have to be carefully designed to avoid primer-dimer amplification and to allow for accurate quantification. Therefore, we took advantage of a validated set of primers containing a set of mismatches that allowed for the annealing and the elongation of the telomeric template, but avoided the formation of primer-dimers (Table 1).

**Figure 3:**
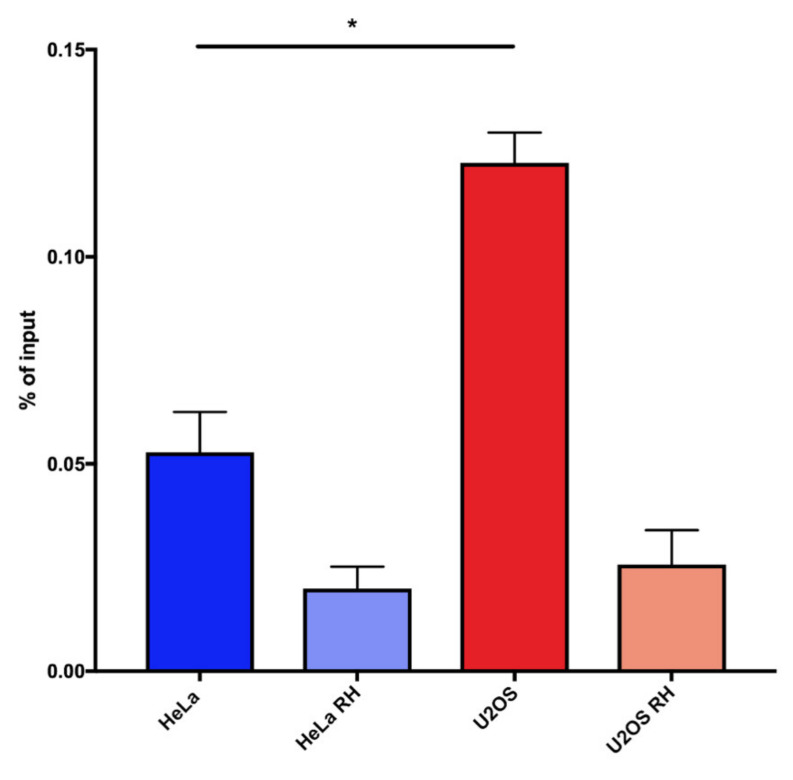
Telomeric DNA:RNA hybrid detection in HeLa and U2OS cells using TeloDRIP-qPCR. Bars and error bars are averages and SEM from four experiments. *p*-values are computed using one-way ANOVA (* *p* < 0.05).

We observed that, also by this procedure, a higher percentage of telomeric repeats were involved in hybrid formation in U2OS cells compared with HeLa cells. The RNAseH-treated controls showed a more effective reduction of the signal compared with dot-blot analysis, indicating that the qPCR detection is more specific for hybrids (Figure 3). The reproducibility and specificity of these results, together with the fact that this protocol is simpler and faster than dot-blot detection, lead us to propose that TeloDRIP-qPCR is a preferable method to detect hybrid forming at telomeres.

## 3. Discussion

Because of their intrinsic characteristics, telomeres are among the genomic loci that display highest levels of replicative stress. DNA:RNA hybrids are one of the features that are responsible for this. The modulation of telomeric DNA:RNA hybrids, and, therefore, of telomeric replicative stress, is particularly important in those cells where telomere maintenance relies on the ALT pathway. In fact, RNAseH1 localizes at the ALT telomeres and its depletion increases the number of DNA:RNA hybrids and, consequently, the markers of replicative stress in those loci. The overexpression of RNAseH1 in ALT cells though, affects telomere maintenance, underlying the fine balance between a recombinogenic environment and a deleterious increase of replication-hindering structures. The important effect of the modulation of telomeric DNA:RNA hybrids in ALT cells could be correlated with increased levels of these structures compared with non-ALT cell lines. This is indicated by DRIP experiments detecting DNA:RNA hybrids at the tiTEL integrated construct and at the subtelomeric regions in a panel of ALT and non-ALT cell lines [7]. However, to the best of our knowledge, whether endogenous telomeric DNA repeats form more frequently DNA:RNA hybrids in ALT cells compared with non-ALT cells has not yet been formally demonstrated. Here, we show that DNA:RNA hybrids involving telomeric repeats are more abundant in ALT cells compared with non-ALT cells. These results are consistent with previous reports [7], and provide a broader view on the landscape of endogenous telomeric DNA:RNA hybrids to include endogenous TTAGGG repeats, which are the most critical part of the telomeres involved in hybrid formation [24,30].

We propose TeloDRIP-qPCR as a new technique for a reliable and fast analysis of telomeric DNA:RNA hybrids. The main advantage of this protocol is the short time required to perform this procedure compared with dot-blot analyses, avoiding the hybridization and exposure steps that often require overnight incubation. In addition, no hazardous reagents, like radionuclides, are needed, reducing exposure to the dangers of ionizing radiations. Finally, TeloDRIP-qPCR does not necessitate large amounts of material: the qPCR analysis of telomeric hybrids shown here required less than one tenth of the material that was used for the dot-blot. This is an important advantage that allows for the analysis of telomeric hybrids starting from small amounts of sample, and enables the study of multiple loci starting from the same DRIP reaction. Finally, the negative controls (the samples treated with RNAse H) seemed to perform better when the same experiments were analyzed by qPCR compared with dot-blot. This could be because of the presence of contaminant RNA that may be detected by the probe, despite the extensive RNAse A treatment. The specificity for the DNA of DNA polymerases used by qPCR may explain the sharper differences observed by TeloDRIP-qPCR.

Therefore, TeloDRIP-qPCR is a useful tool for the study of telomeric DNA:RNA hybrids, and could be applied to many contexts. In fact, ALT tumors are not the only human disease that shows increased levels of TERRA and replicative stress in telomeres. The study of other conditions, such as Werner syndrome or ICF (immunodeficiency, centromeric instability, and facial anomalies) syndrome could benefit from this rapid and easy technique in order to gain better insights into these diseases [24,31]. Another interesting context in which to investigate telomeric hybrid formation is when oncogenes are activated and in aneuploidy [32,33]. Understanding the roles of telomeric R-loops in the telomeric replicative stress induced by these aberrations could unveil unexpected implications for the prevention of malignancy progressions. Moreover, we have widely characterized the transcription of damage-induced long non coding RNAs (dilncRNAs) at the site of DNA double-strand breaks, and their importance in the DNA damage response (DDR) [34,35,36]. In the context of resected double strand breaks, we also demonstrated that dilncRNAs are involved in the formation of DNA:RNA hybrids, which contribute to the recruitment of homologous repair factors and damage repair [37]. In addition, we have reported on the synthesis of dilncRNAs from both strands, both at double strand breaks and at damaged telomeres, including in fibroblasts from Hutchinson–Gilford progeria syndrome (HGPS) patients and in a mouse model [38,39]. Such dilncRNAs can also be involved in the formation of DNA:RNA hybrids. Understanding the dynamics of hybrid formation in these contexts may be important to uncover their possible contributions to DDR in dysfunctional telomeres, the mechanisms underlying human pathologies, and may lead to finding a cure.

## 4. Materials and Methods

### 4.1. Cell Lines and Culture Conditions

HeLa cells (ATCC) were grown in MEM supplemented with 10% fetal bovine serum (FBS), 1% L-glutamine, 1% nonessential amino acids, and sodium pyruvate 1 mM. U2OS cells (ATCC) were grown in McCoy supplemented with 10% fetal bovine serum (FBS) and 1% L-glutamine.

### 4.2. Northern Blot

Northern blot was performed, as described in ref. [40]. Briefly, the total RNA was isolated using the Maxwell RSC simplyRNA tissue kit (Promega, Madison, WI, USA). Then, 10 µg of RNA was run in 1.2% agarose formaldehyde gels. After the run, the gel was treated with 50 mM NaOH for 20 min and transferred onto a Hybond NX neutral nylon membrane (GE Healthcare, Chicago, IL, USA). Hybridization to radiolabeled 5′-(TAACCC)_6_-3′ oligonucleotide was performed overnight at 37 °C. The membranes were washed, exposed to a phosphorimager screen, and subsequently imaged on a Typhoon Imager (GE Healthcare, Chicago, IL, USA).

### 4.3. DNA:RNA Immunoprecipitation (DRIP)

The cells were harvested by scraping and were lysed in 1 mL Tris-EDTA, 0.5% sodium dodecyl sulfate (SDS), and 200 µg/mL proteinase K (Roche, Basel, Switzerland) in presence of RNAseOUT (Invitrogen, Carlsbad, CA, USA) overnight at 37 °C at 350 rpm. Nucleic acids were extracted with phenol/chloroform/isoamyl alcohol (25:24:1 saturated with 10 mM Tris-Cl pH 8.0 and 1 mM EDTA) using MaXtract high density tubes (Qiagen, Hilden, Germany). The samples were precipitated with ethanol, and pellets were spooled out and washed in 70% ethanol. Chromatin was resuspended in 100 µL Tris-EDTA-RNAseOUT and sonicated with a Bioruptor apparatus (Diagenode, Liège, Belgium) at 4 °C (15 s on/30 s off; power: high; cycles: 20, spinning down every five cycles). DNA was quantified at Nanovue, and 10 µg was either treated with 20 U RNAse H (NEB, Ipswich, MA, USA) or mock treated for 5 h at 37 °C at 350 rpm. Four reactions were prepared as follows: 8 µg of chromatin was incubated with 4 µg of S9.6 antibody (MABE 1095, Merck, Darmstadt, Germany) in a binding buffer (10×: 100 mM NaPO_4_ pH 7.0, 1.4 M NaCl, and 0.5% Triton X-100, diluted in Tris-EDTA-RNAseOUT) on a rotating wheel overnight at 4 °C. Before adding the antibody, 1/20 of the volume of the reactions was collected as the input. Immunocomplexes were isolated by incubation with a 1:1 mixture of Dynabeads protein A and protein G (Invitrogen, Carlsbad, CA, USA) for 2 h at 4 °C on a rotating wheel. The beads were washed twice in a binding buffer and were incubated in an elution buffer (50 mM Tris pH 8, 10 mM EDTA, 0.5% SDS) containing 80 µg/mL RNase A (Qiagen, Hilden, Germany) for 30 min at 50 °C at 350 rpm. Elution was performed by adding 560 µg/mL proteinase K (Roche, Basel, Switzerland) and incubating for 45 min at 55 °C at 750 rpm. The supernatants were recovered, the DNA was precipitated, and the four pellets were resuspended in 50 µl Tris-EDTA.

### 4.4. Dot-Blot Detection

Half of the DNA recovered from the DRIP was dot-blotted onto a Hybond N+ nylon membrane (GE Healthcare Chicago, IL, USA) and hybridized to radiolabeled 5′-(GGGTTA)_5_-3′ oligonucleotide overnight at 37 °C. The membranes were washed, exposed to a phosphorimager screen, and subsequently imaged on a Typhoon Imager (GE Healthcare, Chicago, IL, USA).

### 4.5. qPCR Detection

For each well of the qPCR, 0.5 µl samples obtained from the DRIP experiment were used. qPCR detection was performed using a Roche LightCycler 96, the LightCycler 480 SYBR Green I Master mix (Roche, Basel, Switzerland) and the following primer pairs (5′ → 3′): AGGTGCCTTGCTCACAGAGT and GGTTGCATTGCCCTCATTAC for RPL13A and CGGTTTGTTTGGGTTTGGGTTTGGGTTTGGGTTTGGGTT and GGCTTGCCTTACCCTTACCCTTACCC TTACCCTTACCCT for telomeres. The cycles protocol was: preincubation for 5 min at 95 °C; three-step cycling (50 cycles) for 10 s at 95 °C, for 10 s at 60 °C, and for 10 s at 72 °C.

## Figures and Tables

**Table 1:** Telomeric primers used for qPCR detection.

Primer Name	Primer Sequence (5′ → 3′)
Telo FW	CGGTTTGTTTGGGTTTGGGTTTGGGTTTGGGTTTGGGTT
Telo RV	GGCTTGCCTTACCCTTACCCTTACCCTTACCCTTACCCT

## Abbreviations

ALT	Alternative lengthening of telomeres
DDR	DNA damage response
dilncRNA	Damage-induced long non-coding RNA
DRIP	DNA:RNA immunoprecipitation
HGPS	Hutchinson–Gilford progeria syndrome
ICF	Immunodeficiency, centromeric instability and facial anomalies
qPCR	Quantitative polymerase chain reaction
TERRA	Telomeric repeat-containing RNA
